# E-cigarette Awareness among Young Adults: A Pilot Survey Study

**DOI:** 10.7759/cureus.5234

**Published:** 2019-07-25

**Authors:** Vibhav Kanyadan, Latha Ganti

**Affiliations:** 1 Miscellaneous, Wheeler High School, Marietta, USA; 2 Emergency Medicine, Envision Physician Services, Orlando, USA

**Keywords:** vaping, ecigarettes, young adults, tobacco prevention, epidemic, electronic nicotine delivery system, cdc, juul, nicotine, prevalence

## Abstract

Objectives

This study sought to assess: 1) pervasiveness of vaping or electronic cigarette (e-cigarette) use, 2) General understanding of information on vaping or e-cigarette use, 3) Prevalence and respondent awareness of smoking/vaping prevention programs, and 4) Awareness of the harmful effects of e-cigarettes.

Methods

This was a cross-sectional survey of 101 young adults (ages 18-24) in the United States designed to assess the prevalence and knowledge of vaping. Ten questions tested this knowledge and prevalence, some directly (for example, "Which of the following have you used? Select all that apply") and some indirectly (Which of the following pictures corresponds with that of an e-cigarette?). After the results were obtained, the crosstabs showed the percentage of respondents who identified a particular answer for each question.

Results

Most of the surveyants had heard about e-cigarettes (about 88.6% of all respondents). About 55% of the study’s sample attended schools with tobacco prevention programs. Sixty-four percent of the cohort thought that vaping is actually safer than traditional cigarettes. Most (87.4%) of the people were able to identify an e-cigarette when shown a picture of e-cigarettes versus two computer flash-drives and a cigarette. However, 78.2% of respondents were aware of at least one of the effects of nicotine in humans, which corresponds with the number of respondents who were familiar with any of the provided e-cigarette brands (as 71.7% of respondents to question six knew of the six provided brands of e-cigarettes) and the respondents with parents who smoke (as about 75% of respondents said that neither parent smokes). Thirty-eight percent of respondents had used either traditional tobacco products (such as cigarettes, cigars, or chewing tobacco) or e-cigarettes before, with 13% of respondents having used both tobacco and e-cigarettes.

## Introduction

Smoking is a very common addictive behavior among Americans. Each day, about 2000 minors smoke their first cigarette, and according to the Center for Disease Control (CDC), 14% of all adults were cigarette smokers in 2017. Tobacco contains an addictive substance called nicotine. Nicotine is a stimulant that has multiple harmful effects, such as yellowing of nails, sexual impotence, cough, abdominal pain, and lung cancer. Traditional tobacco products, such as cigarettes and cigars, contain over 500 harmful and carcinogenic substances [1]. This may be one reason that many have turned to e-cigarettes (also known as Electronic Nicotine Delivery Systems, or ENDS) under the erroneous belief that e-cigarettes are safer than tobacco. However, e-cigarettes are only shown to be more harmful, as e-cigarettes contain the same nicotine that is found in cigarettes, plus they contain vapor that can produce inflammatory chemicals and impair alveolar macrophages in the lungs, which function to remove organisms and objects that harm the lungs [2].

Smoking is the leading cause of preventable deaths, with over 480,000 deaths per year in the United States [3]. Over 16 million Americans live with a disease caused by smoking, and an additional 41,000 people die from secondhand smoke annually. The Tips from Former Smokers campaign by the Center for Disease Control builds awareness of health damage caused by smoking and encourages smokers to quit, and was effective in causing at least 500,000 current smokers to quit smoking, as well as the US Government’s Smoke Free online program.

In the survey, young adults (18-24) were targeted in order to determine the general notion on how much less harmful e-cigarettes were believed to be. Young adults were additionally targeted because the 2018 National Youth Tobacco Survey showed that vaping had increased by 78% among high-schoolers and 48% among middle-schoolers, showing that current programs are somewhat ineffective in preventing youth from using e-cigarettes [4].

## Materials and methods

A cross-sectional survey of young adults aged 18-24 years in the United States was conducted using the Google Consumer Survey methodology to assess how many respondents were aware of the appearance, properties, and effects of e-cigarettes and vaping. Google Consumer Surveys shows questions across a network of premium online news, reference, and entertainment sites, where they get embedded directly into content, as well as through a mobile app, Google Opinion Rewards. On the web, users answer questions in exchange for access to that content, an alternative to subscribing or upgrading. The user's gender, age, and geographic location are inferred based on anonymous browsing history and IP address. On mobile, users answer questions in exchange for credits for books, music, and apps and users answer demographic questions when first downloading the app. Using this data, Google Consumer Surveys builds a representative sample of hundreds of respondents.

This Google consumer survey was administered in such a way as to garner a validated, representative sample regarding gender, location, and basic demographics of young adults. However, the responses were anonymous, and no protected health information was collected in an identifiable manner. The demographics were collected by the Google survey team, and the specifics were not known to the researcher. Thus, informed consent was not applicable. The background questions targeted the prevalence of smoking, awareness of properties of e-cigarettes, and awareness of their effects. The program knowledge questions targeted the respondents’ knowledge of programs to help them and others quit smoking and vaping, and whether their educational institution ran one of these programs.

## Results

The cohort was comprised of 101 young adults. Sixty percent were female and 40% were male, with all respondents being between 18-24 years old. The United States demographic representation included 33.66% of respondents from the Midwest, 33.66% of respondents from the South, 8.91% of respondents from the Northeast, and 23.76% of respondents from the Western U.S.

**Figure 1:**
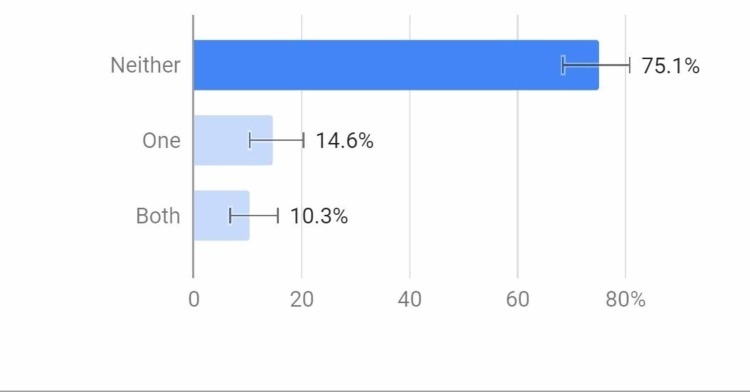
Do your parents smoke?

A total of 75.1% of the population had parents who did not smoke, while 10.3% had both parents smoking at home and 14.6% had one smoker parent (Figure 1).

**Figure 2:**
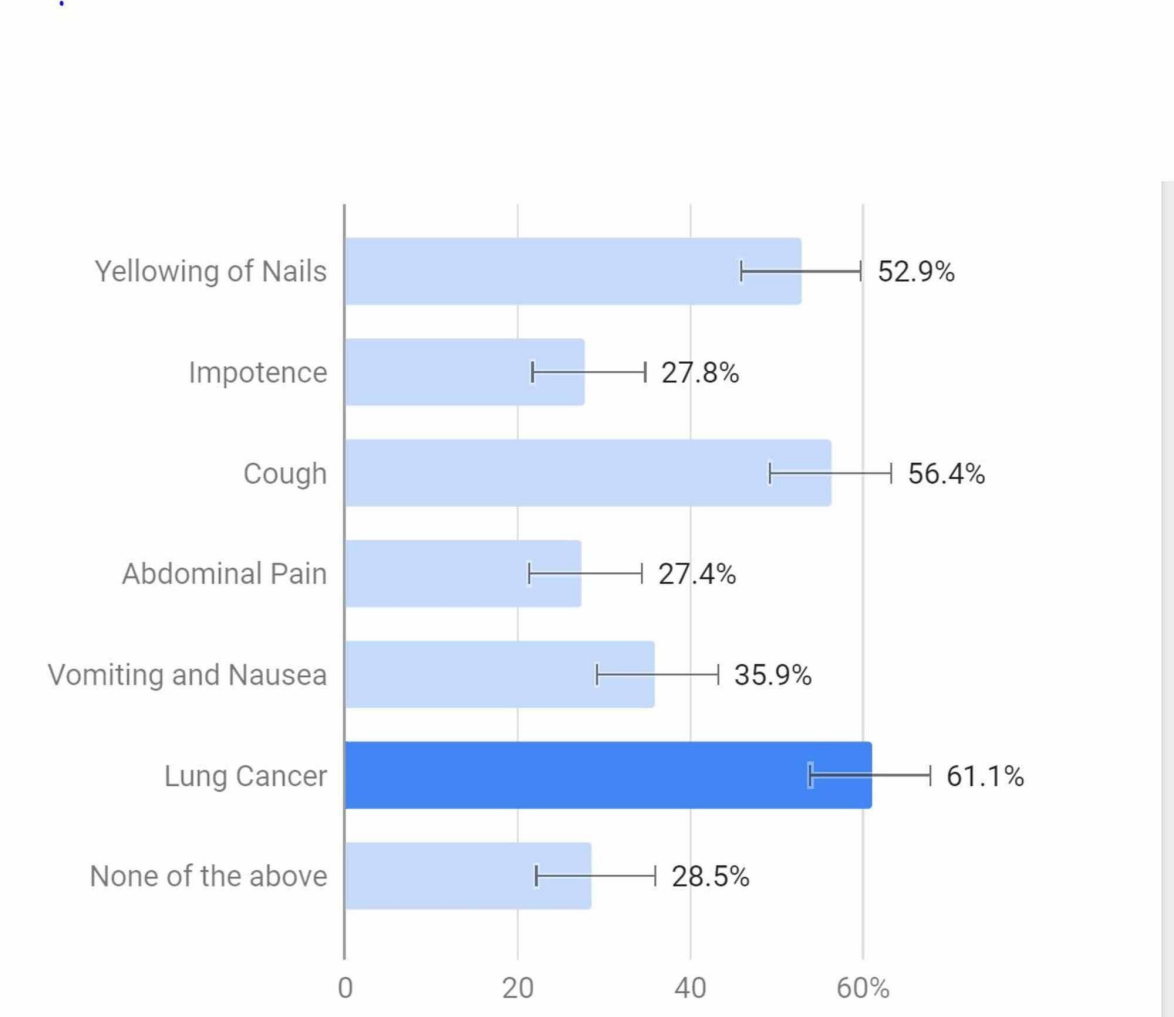
Which of the following effects of nicotine are you aware of?

According to Figure 2, a little over 70% of respondents were aware of at least one of the side effects of nicotine, with a little over 60% of those surveyed were aware of lung cancer as being caused by smoking.

**Figure 3:**
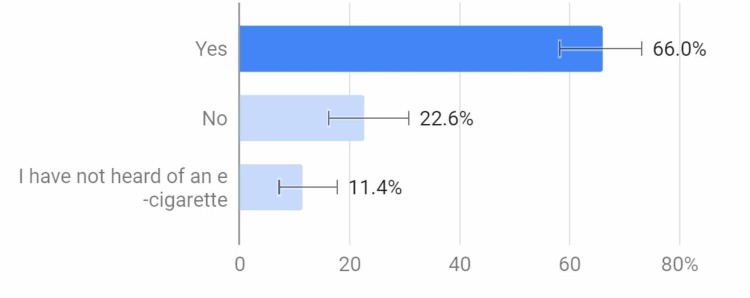
Do you think e-cigarettes and tobacco have similar cancer-causing agents?

Similarly, 66% of respondents thought that tobacco and vapes (e-cigarettes) have similar carcinogens. However, only 89% of the respondents for this question knew what an e-cigarette was (Figure 3).

**Figure 4:**
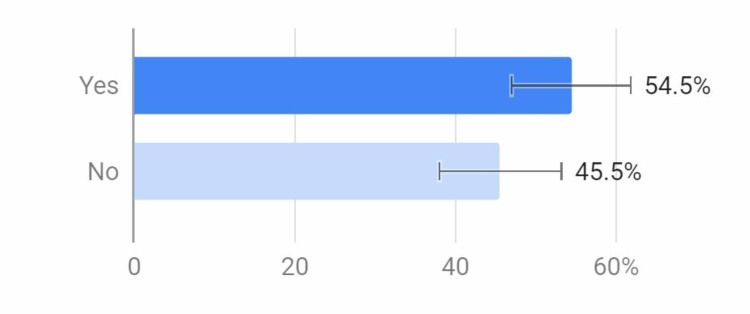
Does your school have a tobacco prevention program?

About 55% of respondents were aware of a tobacco-prevention program at their school, but this may suggest that the other 45% of schools did not offer one, which may affect the number of young adults who adopt the habit of smoking (Figure 4).

**Figure 5:**
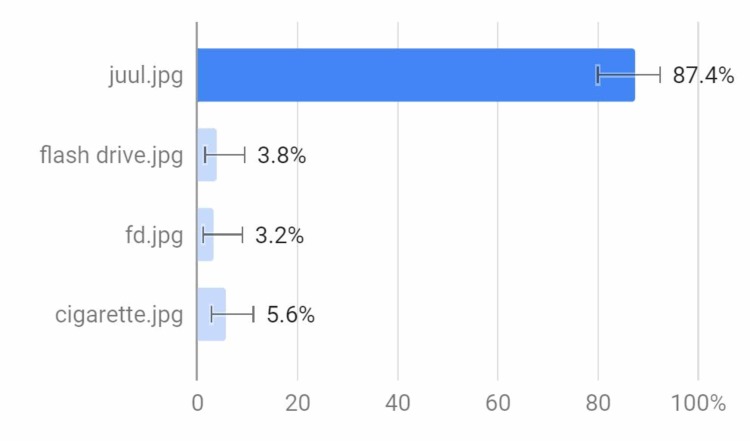
Which one is an e-cigarette?

About 87% of the respondents to this question were able to identify what an e-cigarette looks like. This corresponds with the number of respondents in Figure 3 who did not know what an e-cigarette was (Figure 5). The first image (juul.jpg) was a Juul (a brand of e-cigarette). The second (flash drive.jpg) showed an image of a black flash drive, the third image (fd.jpg) showed an image of a blue flash drive, and the last image was that of a traditional cigarette (cigarette.jpg).

**Figure 6:**
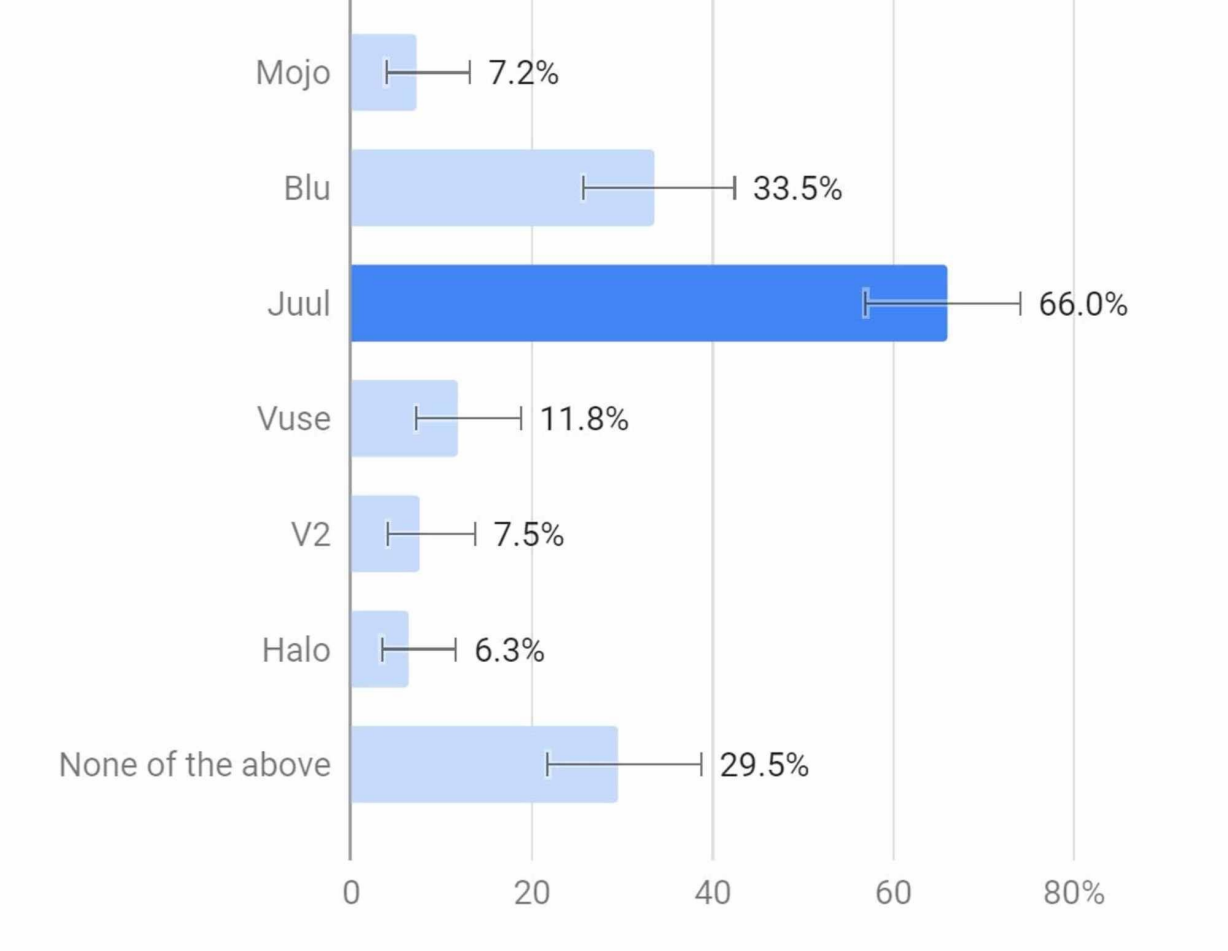
Select which of the following brands of e-cigarette you are familiar with.

Out of all of the respondents, 70.5% were familiar with at least one type of e-cigarette (Figure 6).

**Figure 7:**
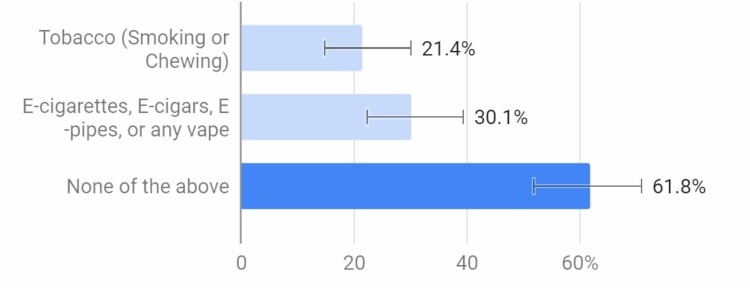
Which of the following have you used?

Out of all of the respondents, 61.8% had never smoked or vaped, while the other 38.2% had used either tobacco or e-cigarettes, or both (Figure 7).

**Figure 8:**
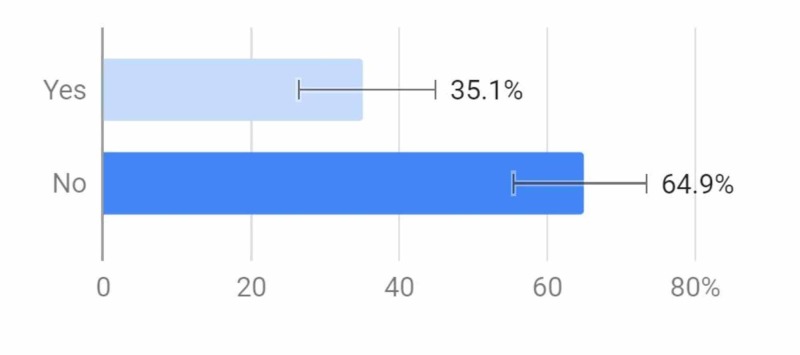
Do you think vaping is safer than using cigarettes?

Out of all of the respondents, about 35% thought that vaping is safer than use of traditional cigarettes (Figure 8).

**Figure 9:**
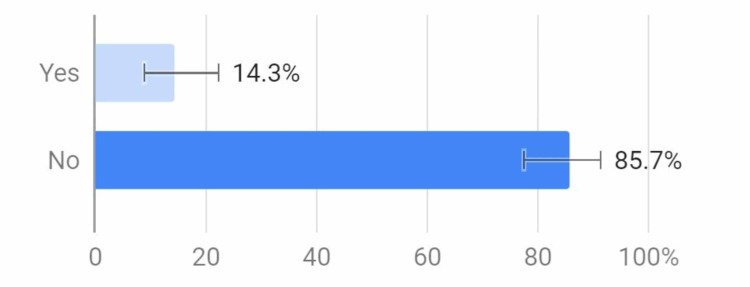
Do you think vaping has a similar odor to cigarette smoking?

Most of the respondents said that e-cigarettes don't have a similar odor to tobacco. This is true, as vapes have particular odors depending on the flavor of aerosol placed inside them (Figure 9).

**Figure 10:**
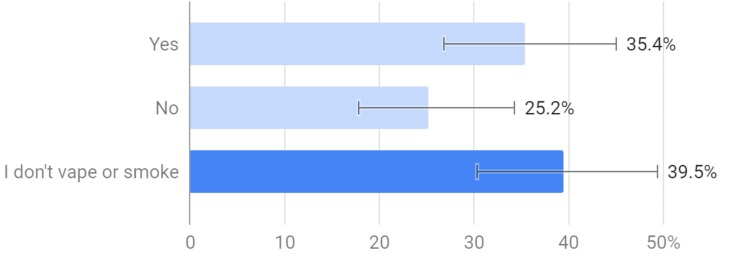
Do you know about the programs and resources available to quit smoking and vaping?

About 75% of respondents either didn't smoke or knew of resources available to quit (Figure 10).

The cross-sectional survey showed that if both of the respondent’s parents smoked, the less likely they were to be aware of any of the harmful effects of nicotine (40% of people of whom both parents smoked or vaped responded “none of the above” for the knowledge of effects question). Additionally, if both of the respondent’s parents smoked, the more likely the young adult was to smoke or vape, and the more likely they were to be unaware of the resources available to quit smoking. Eighty-one percent of respondents who claimed that both their parents smoked also smoked, as only 19.2% of this population selected “I don’t vape or smoke” to Question 9. 

## Discussion

Despite an effort to educate young adults about the dangers of smoking with a freely accessible Smoking fact sheet from the Centers for Disease Control (CDC), only about 70% of young adults from a cross-sectional survey across the United States are aware of at least one harmful effect of nicotine, with the average number of effects per person being close to three out of the six major effects. Although CDC has run the program Tips from Former Smokers since 2012, only 75% of surveyed young adults were aware of programs and resources that were available to them to quit smoking and vaping.

A recent analysis of Twitter feeds shows Twitter users are bonding around, and inquiring about, JUUL on social media [5]. Despite JUUL's branding as a smoking alternative, very few Twitter users mentioned smoking cessation with JUUL. Similar to our survey result, the international Tobacco Control Policy evaluation project (ITC) analyzed survey data from over 4000 adolescents aged 16-19 and found that (ENDS or Electronic Nicotine Delivery Systems) [6].

It can be concluded that there were several factors that led to increases or only a slight decrease in smoking and vaping among young adults. These include lack of awareness of the harmful effects of nicotine, and the belief that vaping is safer than smoking. These results show that parents play an important role in influencing young adults’ attitudes toward smoking, especially if they smoke themselves. Sixty percent of respondents reported that their school ran a tobacco-prevention program. However, the results show that tobacco-prevention programs are not completely effective, as the rate of vaping of students who attended schools that lacked such programs was only 2.6% greater than the rate of vaping of students attending schools with prevention programs. Additionally, the number of smokers and vapers who knew about programs available to quit only rose by 2.2% among those who attended a school with a prevention program compared to those who did not attend a school with a prevention program. Furthermore, a common misconception is that vaping and the use of e-cigarettes is safer than the use of traditional cigarettes. The survey highlights this misconception, as out of the respondents that use e-cigarettes, 65% thought that vapes are safer than tobacco products. Finally, it can be concluded that awareness of resources available to quit was relatively low as well. Half of those who had both parents smoking were unaware of the resources to quit, and in total, one-third of vapers and 22% of smokers were unaware of the resources available to quit.

Smoking and vaping both pose a major public health problem. The estimated incidence of annual worldwide deaths due to smoking is about seven million [7]. The incidence of vaping is increasing [8]. Data from the National Youth Tobacco Survey showed that overall use of tobacco products (including non-combustible products) increased by 38.3% among US high school students in 2018 [9]. The survey also reveals that 4.9 million middle and high school students had used some type of tobacco product in the past 30 days, up from 3.6 million in 2017. This increase was driven entirely by e-cigarette use, as use of other tobacco products actually fell slightly.

Even though all of this information is known and published, the CDC estimates that 45% of adult smokers continue to smoke, or quit and then return to smoking even after reading their guidelines. Nicotine dependence is a growing problem, and made worse by the increasing concentrations of nicotine offered by e-cigarettes. JUUL, the most popular brand of e-cigarettes, commands 75% of market share, and has the highest concentration of nicotine at 5% [10].

There is real concern that the exponential rise in teen vaping rates is creating a generation of nicotine addicts. Indeed, less than 25% of the young adults in this survey had parents who smoked, thanks in large part due to years of anti-smoking measures, especially restriction of smoking in public places such as bars. Progress has been “erased” per the CDC, with over 3.6 million teens using e-cigarettes in 2018.

## Conclusions

This cross-sectional survey of young adults aged 18-24 echoes the existing literature in depicting a widespread use of e-cigarettes, with little awareness of the many associated perils. The results of the cross-sectional survey also point to a large number of people under the impression that e-cigarettes are safer than traditional cigarettes. Despite existing educational campaigns and resources to quit nicotine, the prevalence remains strong, and makes a case for a call to action to prevent an epidemic of nicotine addiction.
